# Dual-Responsive Alginate/PNIPAM Microspheres Fabricated by Microemulsion-Based Electrospray

**DOI:** 10.3390/polym16192765

**Published:** 2024-09-30

**Authors:** Gianluca Ciarleglio, Monica Placido, Elisa Toto, Maria Gabriella Santonicola

**Affiliations:** 1 Department of Chemical Engineering Materials Environment, Sapienza University of Rome, Via del Castro Laurenziano 7, 00161 Rome, Italy; gianluca.ciarleglio@uniroma1.it (G.C.); placido.1772544@studenti.uniroma1.it (M.P.); elisa.toto@uniroma1.it (E.T.); 2 Erbagil s.r.l., Via Luigi Settembrini 13, 82037 Telese Terme, Italy

**Keywords:** natural polymers, poly(N-isopropylacrylamide), electrospray, stimuli-responsive hydrogels, drug delivery

## Abstract

Smart materials for drug delivery are designed to offer a precise and controlled release of therapeutic agents. By responding to specific physiological stimuli, such as changes in temperature and pH, these materials improve treatment efficacy and minimize side effects, paving the way for personalized therapeutic solutions. In this study, we present the fabrication of dual-responsive alginate/poly(N-isopropylacrylamide) (PNIPAM) microspheres, having the ability to respond to both pH and temperature variations and embedding the lipophilic bioactive compound Ozoile. Ozoile^®^ Stable Ozonides is obtained from extra virgin olive oil and acts as an inducer, interacting with major biological pathways by means of modulating the systemic redox balance. The dual-responsive microspheres are prepared by electrospray technique without the use of organic solvents. PNIPAM is synthesized by radical polymerization using the APS/TEMED redox initiators. The microspheres are further optimized with a chitosan coating to enhance their stability and modulate the degradation kinetics of the gel matrix. A comprehensive morphological analysis, Fourier transform infrared (FTIR) spectroscopy, and degradation assays are conducted to confirm the structural stability and pH-responsive behavior of the hydrogel microspheres. A study of the volume phase transition temperature (VPTT) by differential scanning calorimetry (DSC) is used to assess the microsphere thermal response. This research introduces a promising methodology for the development of targeted drug delivery systems, which are particularly useful in the context of oxidative stress modulation and inflammation management.

## 1. Introduction

In recent decades, innovations in polymeric biomaterials have profoundly impacted all aspects of medicine, driving significant transformations in areas such as tissue engineering [1], drug delivery [2], immune engineering [3], and medical device manufacturing [4]. Among these advancements, hydrogel microspheres with stimuli-responsive properties represent a cutting-edge technology in biomedicine and material engineering. Hydrogels are defined as three-dimensional (3D) networks of crosslinked hydrophilic polymers that are capable of absorbing and retaining large amounts of water, and are widely used as drug delivery systems [5,6]. In particular, hydrogel microspheres are spherical particles ranging in size from 1 µm to 1000 µm, which can encapsulate and release various types of drugs in a controlled and sustained manner [7,8], thus improving the bioavailability of the active ingredient [9,10]. Often, these microspheres are endowed with responsive properties to fine tune the release of the bioactive principle, making them smart delivery systems. Smart hydrogels are designed to adapt their chemical and mechanical properties in response to changes in physiological parameters and external stimuli [11], such as variations in temperature [12], pH [13], ultrasound [14], light [15], and magnetic fields [16]. This dynamic responsiveness enables smart hydrogels to provide controlled and targeted therapeutic delivery, making them highly effective for specific medical applications.

Biopolymers, such as alginate and chitosan, are extensively utilized in the formulation of drug delivery systems due to their biocompatibility and because they are easily accessible and renewable raw materials [17]. Alginate is a linear anionic polysaccharide derived from brown algae, which is known for its gelling properties and ability to form hydrogels when crosslinked with divalent cations [18]. Chitosan, on the other hand, is a cationic polysaccharide derived from the exoskeletons of crustaceans, with mucoadhesive properties and antimicrobial activity [19]. Parvaresh et al. synthesized a redox- and pH-responsive magnetic hydrogel based on alginate loaded with doxorubicin [20]. The developed hydrogel has a porous microstructure with a good saturation magnetization value and pH-responsive drug release behavior. Abdellatif et al. developed injectable chitosan hydrogels loaded with 5-fluorouracil for breast cancer treatment, showing extended and controlled drug release over 30 days, effective tumor cell-killing in vitro within 72 h, and significant in vivo tumor inhibition and size reduction, confirmed by histopathological analysis and decreased blood tumor markers [21]. The responsive nature of these materials enhances treatment efficacy and minimizes side effects by providing localized release, optimizing therapeutic outcomes, and reducing systemic toxicity.

Poly(N-isopropylacrylamide) (PNIPAM) is a thermo-responsive polymer that is extensively utilized in biomedical research, particularly for drug delivery systems, due to its biocompatibility and its lower critical solution temperature (LCST) near physiological temperature. The structure of PNIPAM includes hydrophilic amide groups (-CONH-) and hydrophobic isopropyl side chains (-CH(CH_3_)_2_), with a LCST of around 32 °C [22,23]. In solution, PNIPAM is in a sol state at room temperature and transitions to a gel state as it approaches 37 °C [24]. Zhou et al. synthesized mesoporous composite microspheres based on poly-N-isopropylacrylamide-hydroxyapatite (PNIPAM-m-HAP) as a drug carrier for doxorubicin. They demonstrated the biocompatibility, temperature, and pH response of the controlled release system, with characteristics and release kinetics studied in vitro and biocompatibility confirmed by MTT assay [25]. Another advanced technique for the fabrication of microspheres is electrospray (ES), which allows for the control of the size and morphology of the particles [26,27]. This technique is highly effective for the encapsulation and delivery of a diverse range of compounds, including non-water-soluble substances such as Ozoile. Ozoile^®^ Stable Ozonides is a pool of oxygen-rich lipid molecules, obtained through a patented green technology by reacting ozone with the olefin bonds of the fatty acids in organic extra virgin olive oil +OIL^®^ (Erbagil Estate, Telese Terme, Italy). The process leads to the formation of stable ozonides (Criegee mechanism) [28]. Ozoile acts as an inducer capable of interacting with major biological pathways by means of modulating the systemic redox balance [29] with anti-inflammatory [30], antimicrobial [31], and regenerative properties [29]. Using the electrospray technology, it is possible to create microspheres capable of responding to changes in temperature and pH [32,33]. Shamszadeh et al. used the electrospray technique to produce chitosan–alginate core–shell particles for protein delivery [34], showing how bioactivity is dependent on particle size. Ma et al. developed carboxymethylcellulose hydrogel particles loaded with doxorubicin by electrospray for local injection therapy [35]. They demonstrated that the microparticles show a good inhibition of tumor growth due to the sustained release of doxorubicin. In general, most of the studies available in the literature, including those above, have focused on investigating the delivery of hydrophilic compounds by microspheres. The preparation of hydrogel microspheres specifically designed to encapsulate and deliver lipophilic compounds, such as oil-derived molecules, is much less developed.

This study focuses on the fabrication and characterization of dual-responsive alginate and poly(N-isopropylacrylamide) (PNIPAM) (DRAP) microspheres (MSs), designed to respond to both pH and temperature stimuli, for the encapsulation of lipophilic Ozoile. PNIPAM is synthesized by radical polymerization with ammonium persulfate (APS) and N,N,N′,N′-tetramethylethylenediamine (TEMED) as redox initiators. The microspheres are prepared using microemulsion-based electrospray, which offers several advantages, including avoiding the use of organic solvents and the ability to produce microspheres of uniform size and morphology. To further enhance their stability and to control the degradation kinetics of the gel matrix, the microspheres are coated with chitosan, a natural polysaccharide known for its biocompatibility and biodegradability. A comprehensive morphological analysis is conducted using optical microscopy to evaluate the shape and size distribution of the microspheres. Fourier transform infrared (FTIR) spectroscopy is employed to confirm the chemical structure and crosslinking within the hydrogel network. The degradation behavior of the microspheres is assessed through in vitro assays in various media, including phosphate-buffered saline (PBS) at different pH levels. Additionally, the volume phase transition temperature (VPTT), which is one of the main parameters for the microspheres’ responsiveness to temperature changes, is determined using differential scanning calorimetry (DSC). The results of this study demonstrate the successful fabrication of DRAP microspheres with potential applications as drug delivery systems, specifically for non-water-soluble compounds.

## 2. Materials and Methods

### 2.1. Materials

Calcium chloride (CaCl_2_) dihydrate, alginic acid sodium salt, polyethylene glycol sorbitan monooleate (Tween 80), xanthan gum, N-isopropylacrylamide (NIPAM), chitosan (CS) (medium molecular weight, >75% deacetylated), and phosphate-buffered saline (PBS, BioPerformance Certified, pH 7.4, phosphate 0.01 M, NaCl 0.138 M, KCl 0.0027 M) were purchased from Sigma-Aldrich (Milan, Italy). Ammonium peroxodisulfate (APS) and N,N,N′,N′-tetramethyl ethylenediamine (TEMED) were obtained from PanReach AppliChem (Milan, Italy). Ozoile^®^ Stable Ozonides was kindly gifted by Erbagil Estate (Telese Terme, Italy). Deionized water (resistivity 18.2 MΩ·cm) was produced by a Direct-Q3 UV water purification system (Millipore, Molsheim, France) and used in all preparations.

### 2.2. Synthesis of PNIPAM

Radical polymerization was used to synthesize PNIPAM, using APS as an initiator and TEMED as a catalyst [36]. NIPAM (784.44 mM) and APS (30 mM) were added to 45 mL of deionized water. The solution was mixed for 30 min in a cold-water bath using a magnetic stirrer (C-MAG HS7, IKA, Staufen, Germany), and then purged with nitrogen for 30 min to remove oxygen. Subsequently, TEMED (5.93 mM) was added to the solution. The polymerization was carried out at 20 °C using three different polymerization times, 2 h, 4 h, and 6 h. After polymerization, PNIPAM was isolated by precipitation in hot water (T = 60 °C) and purified using ultrapure water. Finally, the polymer was air-dried overnight and then vacuum-dried at 40 °C for 24 h. The average molecular weight (Mn) and polydispersity index (PDI) of PNIPAM synthesized at the above conditions are 26,156 g/mol and 1.8, respectively, as obtained from gel permeation chromatography tests [37].

### 2.3. Microemulsion Preparation

Sodium alginate and the ad hoc synthesized PNIPAM were added at a ratio of 1:1, 3:1, and 5:1 (*w*/*w*) in deionized water, and stirred for 30 min in a cold-water bath. Next, Ozoile (different ratios in range 0–50 wt%), the non-ionic surfactant Tween 80 (5 *w*/*v*%), and the emulsifier xanthan gum (0.25 *w*/*v*%) were added. A stable emulsion was obtained by the high-intensity ultrasound (HIU) method [24] using a handheld ultrasonic homogenizer (UP200Ht, Hielscher Ultrasonics GmbH, Teltow, Germany) at a frequency of 25 kHz, 20 W and 100% power, and for a time of 2.5 min. The HIU method enables the formation of highly stable emulsions: intense shear forces are generated that break down droplets into smaller sizes, leading to a uniform distribution [38]. The emulsions prepared at the above conditions remained stable for over a week. The dynamic viscosity of microemulsions at different Ozoile concentrations was measured at room temperature (25 °C) using a Visco Star plus rotational viscometer (Fungilab, Barcelona, Spain). Viscosity measurements were repeated in triplicates.

### 2.4. Fabrication of Dual-Responsive Alginate/PNIPAM (DRAP) Microspheres

**Figure 1:**
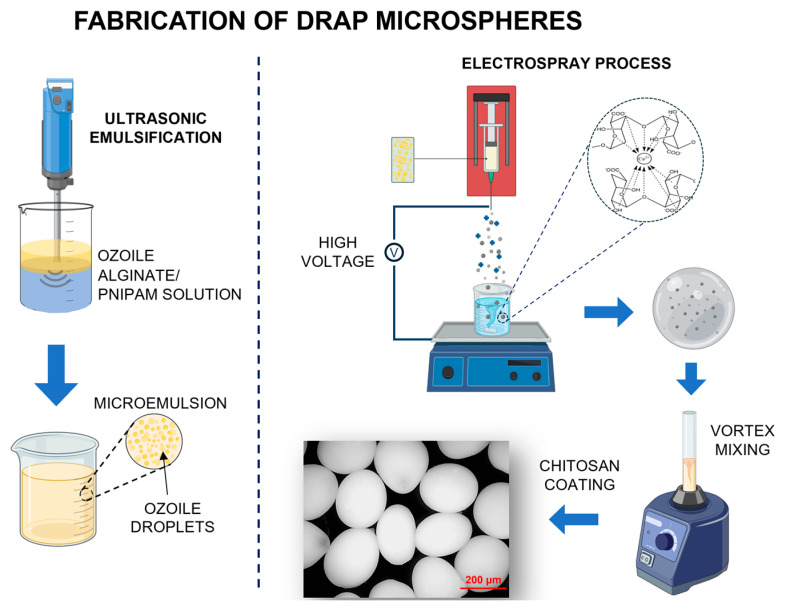
Schematic representation of the fabrication process of DRAP microspheres (MSs) using the electrospray technique. (**Left**): Microemulsion preparation by HIU method. (**Right**): Fabrication of DRAP microspheres by electrospray and coating with chitosan by vortex mixing.

The microspheres were fabricated using the electrospray technique. The microemulsion was placed in a syringe and pushed at a flow rate of 20 mL/h to a stainless steel nozzle (inner diameter 0.311 mm) using an infusion pump (NE-300, New Era Pump Systems, Farmingdale, NY, USA). The spinneret was connected to a high-voltage power supply (CM5, Simco-Ion, Lochem, The Netherlands) at 30 kV. The collecting distance between the spinneret and the CaCl_2_ crosslinking bath (20 wt%) was set at 10 cm. The droplets formed at the tip of the spinneret were ejected as a result of the applied electric field, which overcomes the surface tension. The microdroplets subsequently fell into the crosslinking bath, where ionic gelation occurred. The CaCl_2_ solution was continuously stirred to facilitate the formation of almost spherical microspheres. The physical crosslinking time was set to 30 min. Following the process, the microspheres were placed on filter paper with a 20 µm cut-off (type 589/3, Hahnemühle FineArt, Dassel, Germany) and rinsed several times with ultrapure water to remove any residual CaCl_2_. This step also ensures that only larger microspheres (size > 20 µm) were collected and further used in the investigation. The chitosan coating process was performed by placing the DRAP microspheres in a 0.5 wt% chitosan solution and vortex mixing for 30 min. Next, the microspheres were rinsed three times with ultrapure water to remove the extra chitosan and finally stored in water until use. All experiments were conducted at room temperature (T = 25 °C), with a relative humidity not exceeding 50%. Figure 1 provides a schematic representation of the complete microsphere fabrication process, from the synthesis of PNIPAM to the use of the electrospray technique for the formation of DRAP MSs.

### 2.5. Cloud Point Determination

To evaluate the thermal response of the in-house-made PNIPAM, the cloud point of four solutions prepared using different polymer concentrations (0.5 to 3 wt%) and crosslinking times (2 h, 4 h, and 6 h) was investigated. The temperature of the water bath was adjusted to start at 25 °C and increased at a rate of 2 °C/h. Cloud points were determined using a Dino-Lite AM7915MZT optical microscope (AnMo Electronics Corporation, New Taipei City, Taiwan).

### 2.6. Morphological Characterization

A DinoLite AM7915MZT optical microscope was used to evaluate the microspheres’ morphology, shape, and size. The diameter size distribution of the microspheres was evaluated with ImageJ software (version 1.53) [39].

### 2.7. Swelling Behavior

The hydrogel microspheres were completely hydrated in deionized water at room temperature (T = 25 °C) before being dried at 50 °C for 18 h to determine the swelling ratio and water content. The following equations were used to determine the swelling ratio and water content [40]:

(1) $$Swelling\;Ratio=\frac{W_{s}}{W_{d}}$$

(2) $$\%\;water\;content=\frac{\left(W_{s}-W_{d}\right)}{W_{s}}\times 100$$

where W_s_ is the weight of the hydrogel in its equilibrium swelling state, and W_d_ is the weight of the fully dried hydrogel. The test was repeated on five samples for each microsphere type and the results averaged.

### 2.8. Thermal Characterization

The volume phase transition temperature (VPTT) of the PNIPAM solutions and the bound water fraction (X_BW_) of the DRAP microspheres were determined by differential scanning calorimetry. A DSC 8500 instrument (PerkinElmer, Waltham, MA, USA) calibrated with high-purity indium and tin was used. DSC samples were sealed in aluminum pans with lids and measurements were performed under a constant flow of nitrogen (40 mL/min). An identical empty cell was taken as a reference. To determine the VPTT of the PNIPAM solutions, the samples (three replicates, ∼10 mg) were analyzed in the temperature range of 25–50 °C with a heating rate of 10 °C/min. To determine the VPTT of the DRAP microspheres, the swollen hydrogel samples (three replicates, ∼25 mg) were subjected to a thermal cycle consisting of three steps: heating from 10 °C to 50 °C with a scan rate of 10 °C/min, holding at 50 °C for 1 min, and cooling from 50 °C to 10 °C with a scan rate of 10 °C/min. For X_BW_ determination, the swollen microspheres (three replicates, ∼25 mg) were analyzed in the temperature range from −30 °C to 30 °C with a heating rate of 10 °C/min. X_BW_ was calculated with the following equation [41]:

(3) $$X_{BW}=X_{TW}-\frac{Q_{endo}}{Q_{f}}$$

where Q_endo_ is the heat of fusion of freezable water in the microspheres, which is obtained from the DSC thermogram (J/g) by calculating the peak area with PerkinElmer Thermal Analysis software (version 13.3.1.0014). Q_f_ indicates the heat of fusion of pure water (333 J/g) [42] and X_TW_ is the total water fraction calculated according to the following equation:

(4) $$X_{TW}=1-\frac{M_{DRY}}{M_{TOTAL}}$$

where M_DRY_ indicates the dry mass and M_TOTAL_ the total wet mass of the microspheres.

### 2.9. ATR-FTIR Spectroscopy

Fourier transform infrared (FTIR) spectra of DRAP MSs were recorded using a Nicolet Summit Spectrometer (Thermo Fisher Scientific, Waltham, MA, USA) with a ZnSe ATR accessory. IR spectra were acquired in the wavenumber region of 4000–800 cm^−1^, with a resolution of 4 cm^−1^. Each spectrum was obtained after averaging 64 scans.

### 2.10. Degradation Tests

Degradation tests were performed in deionized water and in PBS solutions with different pH values (2, 4, 6, 7.4), monitoring the microspheres’ behavior every hour for the first 4 h and after 24 h. DRAP MSs loaded with 30 wt% of Ozoile, with and without chitosan coating, were investigated. Tests were repeated at 25 °C and at physiological temperature, 37 °C. To understand the phenomenon of degradation as the pH changes, the deprotonation rate of carboxyl groups was calculated using the Henderson–Hasselbalch equation [43]:

(5) $$\%\;deprotonation=\frac{10^{\left(pH-{pK}_{A}\right)}}{10^{\left(pH-{pK}_{A}\right)}+1}\times 100$$

where pK_A_ is the acid dissociation constant of the carboxylic group. This formula expresses the relationship between the pH of the environment and the percentage of deprotonated carboxylic groups at that specific pH.

## 3. Results and Discussion

### 3.1. Thermal Response of PNIPAM

The synthesis of PNIPAM was carried out by radical polymerization, using APS as the initiator and TEMED as the catalyst. Three different polymerization times were used: 2 h, 4 h, and 6 h. To evaluate the thermal response of PNIPAM at the different polymerization times, two experiments were performed: cloud point evaluation of PNIPAM solutions (0.5 to 3 wt%) and DSC analysis with VPTT determination for PNIPAM solutions at 2 wt%.

The cloud point measurements for PNIPAM solutions at various concentrations (0.5–3 wt%) and different times of polymerization (t_p_), 2 h, 4 h, and 6 h, are summarized in Table 1.

The cloud point of the PNIPAM solutions showed no statistically significant changes as the concentration and polymerization time changed. Under the conditions tested, the cloud point remained relatively stable within the temperature range of 32–34 °C, and the observed variations were within the range of experimental variability.

**Figure 2:**
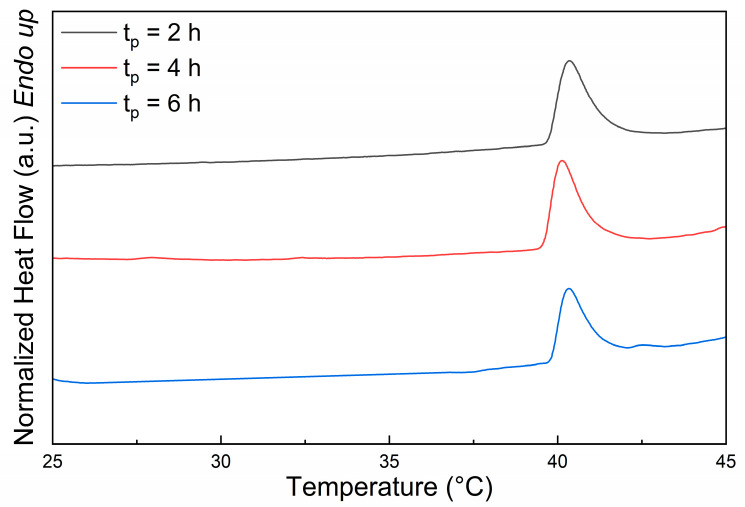
DSC thermograms of aqueous solutions (2 wt%) of PNIPAM synthesized by redox-initiated radical polymerization with different times (t_p_).

DSC measurements were carried out to determine the VPTT of the PNIPAM solutions. The thermograms were obtained by analyzing the polymer solutions (2 wt% in water) in the temperature range 10–50 °C with a heating rate of 10 °C/min (Figure 2).

Table 2 shows the values of the VPTT and the enthalpy (∆H) of PNIPAM solutions at 2 wt% for different t_p_. These results indicate that the VPTT is similar (approximately 40 °C) for all considered samples, with minimal variation between different polymerization times.

Considering the results regarding the cloud points and the VPTTs of the PNIPAM solutions, no statistically significant differences emerged for PNIPAM obtained at different polymerization times (2 h, 4 h, 6 h). Since there is no significant advantage in further extending the polymerization time, for the preparation of the DRAP microspheres, it was decided to use PNIPAM synthesized with a 2 h polymerization time.

### 3.2. Fabrication of DRAP Microspheres and Morphological Analysis

DRAP microspheres containing Ozoile (0–50 wt%) were fabricated using the electrospray technique starting from highly stable microemulsions. The microspheres were ionically crosslinked in a CaCl_2_ bath (20 wt%) for 30 min. The microemulsions were produced via high-intensity ultrasound (HIU) and exhibited stability for over one week. The dynamic viscosities of the microemulsions were measured by rotating cylinder viscometry at varying Ozoile concentrations (0–50 wt%), and they are reported in Table S1.

The following electrospray parameters were used: needle with an inner diameter of 0.311 mm, applied voltage of 30 kV, flow rate of 20 mL/h, and collecting distance of 10 cm. The process was conducted at a temperature of 25 °C, with a relative humidity < 50%.

**Figure 3:**
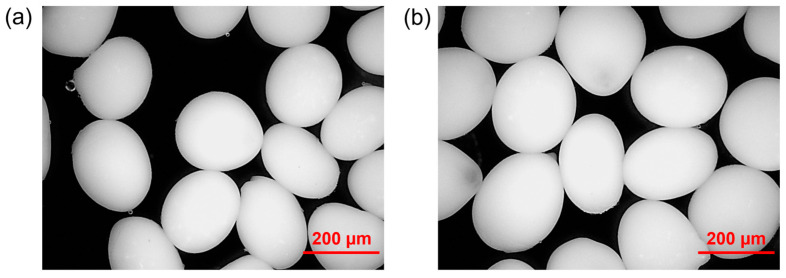
Optical microscopy images of hydrated DRAP microspheres containing Ozoile (30 wt%) (**a**) without coating and (**b**) with chitosan coating.

To assess the morphology of the microspheres, an optical microscope analysis was conducted (Figure 3). The microsphere size was investigated using the ImageJ software, starting from 250 sample images for each type of microsphere. For the analysis, the images were first binarized and then the average diameter of the microspheres was measured using the “Analyze particles” tool.

The results in terms of mean diameter and kurtosis are shown in Table 3. The data indicate that all types of microspheres exhibit a mean diameter of less than 450 μm. The results also indicate that the mean diameter of the chitosan-coated microspheres is greater than that of the corresponding uncoated microspheres for Ozoile concentrations above 20 wt%. This is in agreement with an increase in the microspheres’ size due to the formation of a chitosan shell on the outside the DRAP microspheres.

A negative kurtosis value indicates a flat and widely dispersed distribution, whereas a positive value suggests a peaked distribution. As illustrated in Table 3, the microspheres exhibiting the highest kurtosis values are those containing 30 wt% Ozoile, with kurtosis values of 3.12 and 1.88 for the microspheres without and with chitosan coating, respectively. The microspheres containing 30 wt% Ozoile exhibited the best characteristics in terms of size and homogeneity, with average diameters of about 207 μm and 216 μm for the uncoated and chitosan-coated MSs, respectively. All other DRAP microspheres with Ozoile presented a wider distribution in size, as highlighted by their large standard deviations.

### 3.3. Swelling Properties

**Figure 4:**
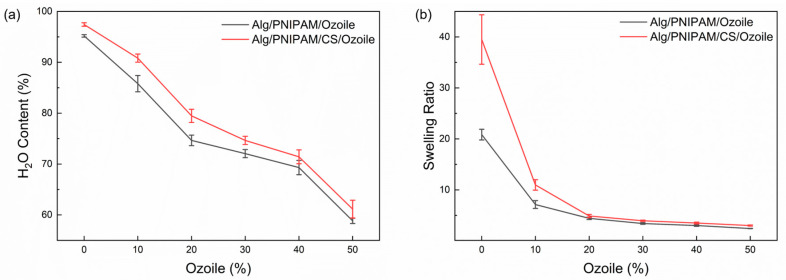
(**a**) Water content and (**b**) swelling ratio of DRAP microspheres at different Ozoile concentrations (0–50 wt%). Test performed in water at T = 25 °C.

Figure 4 shows the water content (%) and the swelling ratio of DRAP microspheres containing Ozoile (0–50 wt%) at room temperature (T = 25 °C). All types of microspheres exhibit a water content above 50%, with large values that are similar to those of biological tissues [44].

In particular, microspheres based on sodium alginate and PNIPAM exhibit a higher water content (greater than 90%) than those with Ozoile. Ozoile, due to its hydrophobic nature, acts as a water repellent, thereby reducing the degree of swelling of the microspheres. This phenomenon can be attributed to the presence of strongly hydrophilic groups, namely hydroxyl (-OH) and carboxyl (-COOH), within the alginate polymer chain [45], as well as the hydrophilic amide group (-CONH) in the PNIPAM polymer chain below the VPTT [46].

The presence of chitosan has been observed to increase both parameters for all types of microspheres. This phenomenon may be attributed to the hydrophilic nature of chitosan, which contains hydroxyl (-OH), amine (-NH_2_), and carboxylic (-COOH) functional groups. These groups may be responsible for the microspheres’ increased affinity for water and their ability to absorb excess water molecules [47].

### 3.4. FTIR Analysis

**Figure 5:**
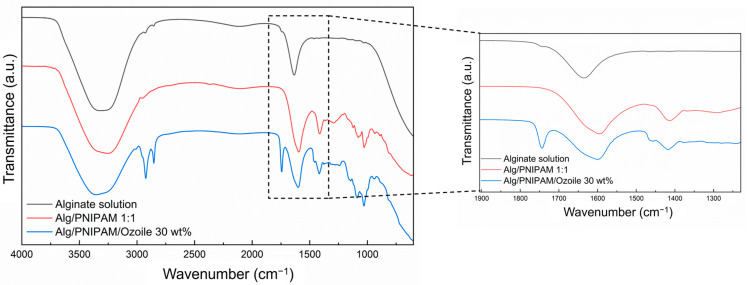
FTIR spectra of pure alginate solution, alginate/PNIPAM microspheres (1:1 ratio), and DRAP microspheres containing Ozoile (30 wt%).

The FTIR analysis was performed to investigate the chemical structure and the physical crosslinking of the hydrogel matrix of the DRAP microspheres, as well as their loading with Ozoile. Figure 5 shows the spectra of the pure sodium alginate solution, of the empty DRAP MSs, and of the microspheres containing Ozoile at 30 wt%. Compared with the solution of sodium alginate, there is a noticeable shift in the stretching bands of the -COO^−^ group (from 1637 to 1597 cm^−1^) in the MS spectra due to the formation of an ionic bond between the Ca^2+^ ions and the deprotonated carboxyl groups of alginate [48]. This shift confirms that physical crosslinking in the DRAP microsphere matrix has occurred.

**Figure 6:**
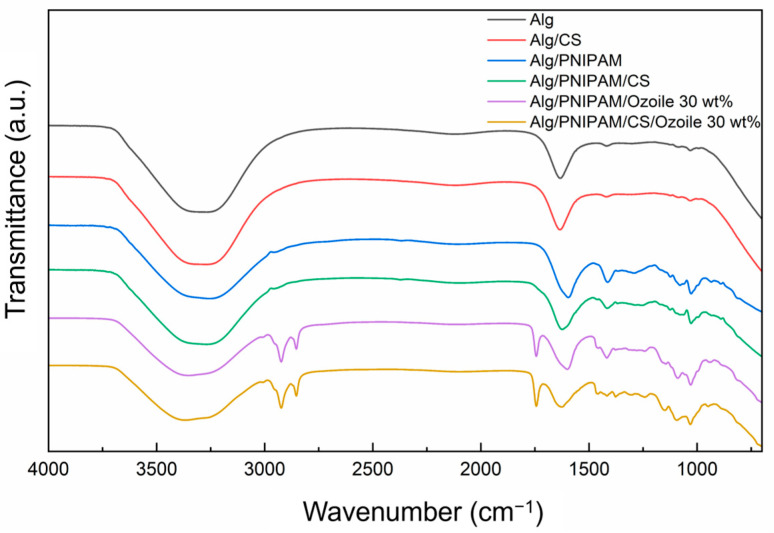
FTIR spectra of alginate microspheres, alginate/PNIPAM microspheres (1:1 ratio), and DRAP microspheres containing Ozoile (30 wt%) with and without chitosan coating.

The FTIR analysis was also used to assess the presence of Ozoile and PNIPAM in the microspheres. Figure 6 shows the spectra of alginate microspheres, alginate/PNIPAM microspheres (1:1 ratio), and DRAP microspheres containing 30 wt% Ozoile with and without chitosan coating. All types of microspheres, with and without chitosan coating, show a broad absorption band at about 3700 cm^−1^ and 3000 cm^−1^ corresponding to O-H and N-H stretching [49]. The characteristic peak at 1602 cm^−1^ confirms the presence of PNIPAM and sodium alginate. This peak can be assigned to the amide band I (C=O stretching) of PNIPAM and to the asymmetric and symmetric stretching of the -COO^−^ group of alginate [49,50]. Moreover, the presence of alginate is confirmed by the peaks at 1416 and 1029 cm^−1^, which are characteristic of the CO-C vibration of the groups in the guluronic units [51]. The FTIR spectra for the microspheres containing Ozoile present two absorption bands around 2920 and 2850 cm^−1^, which originate from the asymmetric and symmetric stretching vibrations of the methylene (-CH_2_) and methyl (-CH_3_) groups, respectively [52].

### 3.5. Thermal Properties of DRAP Microspheres by DSC

**Figure 7:**
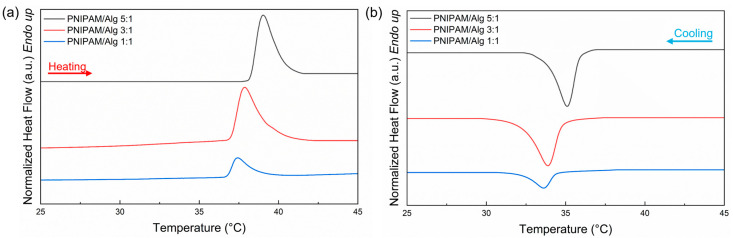
DSC thermograms of DRAP microspheres prepared with different ratios of PNIPAM and alginate during (**a**) heating and (**b**) cooling scan.

Differential scanning calorimetry (DSC) was used to analyze the volume phase transition temperature (VPTT) and the bound water fraction (X_BW_) of the DRAP microspheres. Figure 7 shows the thermograms related to the heating and cooling cycles performed to investigate the thermal response of microspheres with different ratios of PNIPAM and sodium alginate (5:1, 3:1, 1:1). The VPTT values and the corresponding enthalpy (ΔH) values were calculated and are listed in Table 4. For all types of microspheres, there is a large thermal response and the phenomenon is reversible. Furthermore, VPTT shows hysteresis between heating and cooling due to the propensity of PNIPAM to form a metastable state [53,54]. Although the 3:1 and 5:1 ratios showed a good thermal response, the solutions were very viscous and not processable by electrospraying. Therefore, it was decided to adopt the 1:1 ratio of PNIPAM and sodium alginate for the production of the mixed DRAP microspheres.

**Figure 8:**
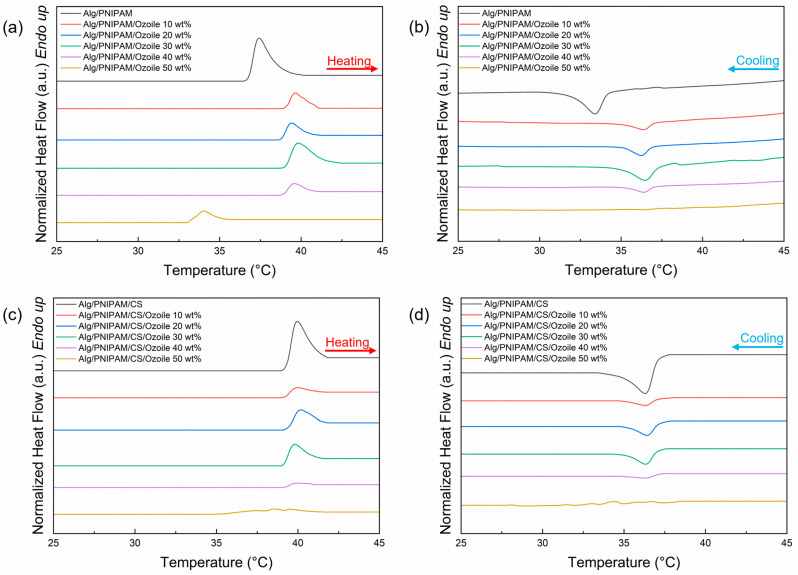
DSC thermograms for VPTT evaluation of DRAP microspheres containing Ozoile (0–50 wt%): (**a**,**b**) microspheres without coating and (**c**,**d**) with chitosan coating.

Figure 8 shows the thermograms for the heating and cooling step of the DSC runs on microspheres containing Ozoile (0–50 wt%) with and without chitosan coating. The VPTT values and the corresponding enthalpy values (ΔH) were calculated and are listed in Tables S2 and S3, respectively. Microspheres containing Ozoile up to 40 wt% show a good thermal response around 39.5–40 °C, and there are no notable differences between the microspheres with and without chitosan coating. The difference between the VPTTs obtained from the heating and cooling scans is due to overheating, subcooling of the phase transition, and temperature hysteresis in the DSC test. Microspheres with an Ozoile concentration of 30 wt% showed the best thermal response in terms of enthalpy change value (Tables S2 and S3). In contrast, microspheres with the highest Ozoile concentration (50 wt%) showed a very limited thermal response. The presence of the lipophilic Ozoile compound appears to hinder the response of the PNIPAM/alginate hydrogel network during temperature changes around the VPTT. This could be due to a hydrophobic interaction between Ozoile and PNIPAM in its collapsed (hydrophobic) state, which results in limited movement of the PNIPAM chains.

**Figure 9:**
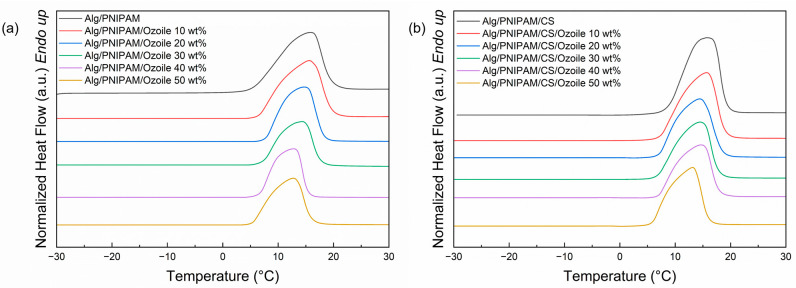
DSC thermograms for X_BW_ evaluation of alginate/PNIPAM microspheres at different Ozoile concentrations (0–50 wt%): (**a**) without coating and (**b**) with chitosan coating.

Figure 9 shows the thermograms obtained for DRAP microspheres at different concentrations of Ozoile (0–50 wt%), with and without chitosan coating, measured in the range of −30–30 °C to determine the fraction of bound water (X_BW_) to the hydrogel matrix. Table 5 shows the values of the bound water fraction (X_BW_) and the melting enthalpies (ΔH_f_) of DRAP MSs, as determined by the DSC analysis.

The endothermic peak is in the range between 5 °C and 20 °C and is attributable to the presence of free water in the hydrogels. There is an increase in the fraction of bound water (X_BW_) as the concentration of Ozoile increases. This may be due to the increased formation of hydrogen bonds with the -COO^−^ groups of the ozonated oil.

### 3.6. Degradation Tests of DRAP Microspheres

Degradation tests were conducted on the Ozoile-containing DRAP microspheres, with and without chitosan coating, to assess the thermo- and pH-responsive nature of the microspheres. Tests were carried out at varying pH values (2, 4, 6, 7.4) and at two different temperatures: 25 °C (room temperature) and 37 °C (physiological temperature). The tests were conducted on the DRAP microspheres containing 30 wt% of Ozoile. In fact, based on the DSC results, these microspheres exhibited the best thermal response among those containing Ozoile, with a larger enthalpy change during volume phase transition (Tables S2 and S3), and were therefore selected for the degradation tests.

**Figure 10:**
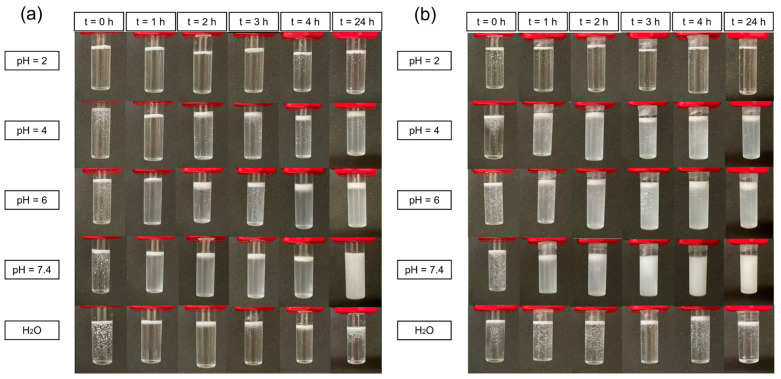
Degradation tests for uncoated DRAP microspheres containing Ozoile (30 wt%). Tests performed in PBS with different pH values and in pure water at temperatures of (**a**) 25 °C and (**b**) 37 °C.

Figure 10 shows the degradation tests of DRAP MSs containing 30 wt% Ozoile at different pH values and at temperatures of 25 °C (Figure 10a) and 37 °C (Figure 10b).

The entire degradation process of alginate is driven by the carboxyl group -COOH of its mannuronic (M) block [40]. It can be observed that the degradation of the microspheres occurs more slowly in an acidic environment. In particular, degradation does not occur at pH 2, as the pH is below the pKa of alginate (3.6). The carboxyl groups remain in their protonated -COOH form, preventing the disintegration of the polymer network.

**Figure 11:**
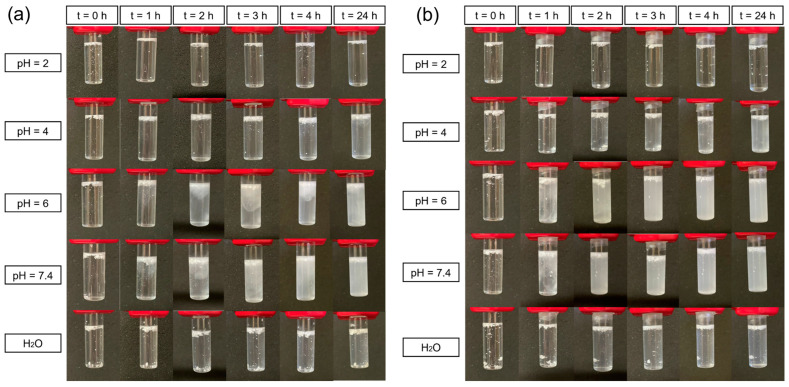
Degradation tests for chitosan-coated DRAP microspheres containing Ozoile (30 wt%). Tests performed in PBS with different pH values and in pure water at temperatures of (**a**) 25 °C and (**b**) 37 °C.

Using the Henderson–Hasselbalch equation, the deprotonation percentage at the various pH levels was calculated. At pH 4, degradation occurs because pH > pKa of alginate (pKa = 3.6), resulting in 71.5% of the acid groups being deprotonated. In this condition, an electrostatic repulsion among the polymer chains, carrying the negative -COO^−^ groups, occurs, leading to the release of Ozoile. At pH 6, the degradation process is still driven by the interactions of the -COO^−^ groups (99.6% of carboxylic groups are deprotonated) causing electrostatic repulsion between the polymer chains, which in turns leads to the dispersion of the lipophilic Ozoile in water. At pH 7.4, there is a further increase in degradation due to the even larger extent of deprotonation, reaching a value of 99.98%. Additionally, the presence of Na^+^ ions in the phosphate-buffered saline (PBS) solution initiates an ion exchange process with the Ca^2+^ cations that are bound to the carboxyl groups of the M blocks of alginate, leading to dissolution. This phenomenon does not occur in ultrapure water due to the absence of salts or in solution at pH 2 because the carboxyl groups are in their protonated form (-COOH). When considering pH values of 4, 6, and 7.4, an acceleration of the degradation process of the DRAP microspheres is noted at higher temperature (37 °C) with respect to the lower 25 °C in both the cases of uncoated (Figure 10) and chitosan-coated (Figure 11) microspheres. This is because, at 37 °C, the microspheres are closer to their VPTT (Tables S2 and S3), which leads to a more rapid collapse at physiological temperature than at room temperature. The VPTT represents the temperature at which the polymer system undergoes a phase transition from a hydrated/swollen state to a dehydrated/collapsed state, which promotes the collapse and consequent release of the microspheres’ content.

Figure 11 shows the time evolution of the degradation tests for the chitosan-coated DRAP microspheres with 30 wt% of Ozoile at different pH values and temperatures (25 °C and 37 °C). The presence of the chitosan coating does not result in significant differences compared to the test performed on uncoated microspheres. For the coated microspheres, a slight slowing-down of the degradation process can be observed at both 25 °C and 37 °C. This might be due to the opposite charges characteristic of alginate and chitosan creating a strong electrostatic attraction between the two polymers. In acidic environments, this attractive force is stronger due to the protonation of the amine groups of chitosan. At pH 4 and 6, which are above the pKa of alginate (3.6) but below that of chitosan (6.5), the amine groups of chitosan are protonated (-NH_3_^+^), while the carboxyl groups of alginate are in their -COO^−^ form. At pH 7.4, both groups are deprotonated, with deprotonation degrees of 99.98% and 88.81% for alginate and chitosan, respectively.

The thermo-responsive nature of the DRAP MSs, uncoated and chitosan-coated, is highlighted by comparing the results of the tests performed at 25 °C and 37 °C (Figure 10 and Figure 11). For both types of microspheres, it can be seen that the dissolution of the polymer mesh is faster at the temperature of 37 °C. In fact, at 37 °C (above the cloud point), PNIPAM is in its hydrophobic and collapsed state. The collapse of the polymer network accelerates the release of Ozoile in the aqueous PBS solutions.

## 4. Conclusions

Dual-responsive hydrogel microspheres based on alginate and PNIPAM polymers, and containing the lipophilic active principle Ozoile, were successfully fabricated by an electrospray process. The thermo-responsive PNIPAM was synthesized using radical polymerization, ensuring the desired properties for the dual-responsive system. DRAP microspheres were fabricated by microemulsion-based electrospray technology using water as solvent, followed by ionic gelation. The system parameters, particularly the ratio of alginate to PNIPAM, were optimized to achieve a processable solution that could be effectively electrosprayed. This was crucial for ensuring the successful fabrication of the microspheres. All produced microspheres exhibited a water content greater than 50%. Notably, an increase in the concentration of Ozoile resulted in a decrease in water content and swelling ratio, indicating a correlation between the Ozoile concentration and the microsphere hydration properties. The thermal response in terms of volume phase transition temperature (VPTT) of the DRAP microspheres was evaluated by DSC. Microspheres containing 30 wt% Ozoile showed the largest thermal response, making them particularly interesting for temperature-sensitive applications. Degradation tests conducted at different pH values and temperatures demonstrated the dual responsiveness of the microspheres. DRAP microspheres are resistant to highly acidic environments (pH = 2), but dissolve at higher pH levels in few hours. The chitosan coating plays a modulating role by influencing the dissolution behavior at different pH values. Overall, the microspheres fabricated in this work showed an effective response to changes in pH and temperature, confirming their stimulus-responsive nature and ability to degrade and deliver their active content. This research introduces a promising methodology for the development of an advanced drug delivery system for non-water-soluble compounds, such as Ozoile, with potential applications in managing oxidative stress and inflammation in various biomedical contexts.

## Figures and Tables

**Table 1:** Cloud points of aqueous solutions of PNIPAM obtained with polymerization times (t_p_) of 2 h, 4 h, and 6 h. PNIPAM concentration in range 0.5–3 wt%.

Concentration (wt%)	Cloud Point (°C)		
	t_p_ = 2 h	t_p_ = 4 h	t_p_ = 6 h
0.5	34.14 ± 0.10	33.92 ± 0.26	33.60 ± 0.29
1	33.88 ± 0.27	33.92 ± 0.37	34.27 ± 0.32
2	34.10 ± 0.87	33.82 ± 0.86	32.36 ± 0.66
3	33.76 ± 0.62	33.32 ± 0.95	32.16 ± 0.91

**Table 2:** VPTT and ∆H values of PNIPAM solutions (2 wt%) with different polymerization times (t_p_).

t_p_ (h)	VPTT Heating (°C)	∆H Heating (J/g)
2	40.33 ± 0.21	0.89 ± 0.09
4	40.12 ± 0.26	0.85 ± 0.12
6	40.31 ± 0.73	0.67 ± 0.05

**Table 3:** The mean diameter and kurtosis of DRAP microspheres at different Ozoile concentrations (0–50 wt%) without and with chitosan coating.

	No Chitosan Coating		Chitosan Coating	
Ozoile (wt%)	Diameter (μm)	Kurtosis	Diameter (μm)	Kurtosis
0	423.12 ± 133.07	0.54	443.22 ± 124.07	0.88
10	412.26 ± 290.05	−1.33	398.26 ± 247.60	−1.32
20	265.70 ± 220.26	0.94	375.10 ± 219.62	−0.93
30	206.60 ± 155.38	3.12	215.93 ± 161.07	1.88
40	234.54 ± 165.13	2.47	278.23 ± 243.93	−0.71
50	272.62 ± 155.58	2.64	353.54 ± 336.43	−0.71

**Table 4:** VPTT, T_onset_, and ∆H of DRAP microspheres with different ratios of PNIPAM and alginate obtained from DSC analysis.

PNIPAM/Alginate	T_onset_ Heating (°C)	VPTT Heating (°C)	∆H Heating (J/g)	T_onset_ Cooling (°C)	VPTT Cooling (°C)	∆H Cooling (J/g)
5:1	38.46 ± 0.37	39.27 ± 0.42	1.61 ± 0.07	35.69 ± 0.14	34.84 ± 0.20	−1.57 ± 0.03
3:1	36.96 ± 0.26	37.68 ± 0.33	1.25 ± 0.37	34.60 ± 0.12	33.78 ± 0.08	−1.23 ± 0.24
1:1	36.92 ± 0.22	37.55 ± 0.22	0.46 ± 0.03	34.40 ± 0.18	33.61 ± 0.17	−0.50 ± 0.04

**Table 5:** Bound water fraction (X_BW_) and melting enthalpy (ΔH_m_) values of DRAP microspheres obtained from DSC analysis.

	No Chitosan Coating		Chitosan Coating	
Ozoile (wt%)	∆H_m_ (J/g)	X_BW_ (%)	∆H_m_ (J/g)	X_BW_ (%)
0	304.94 ± 7.25	3.84 ± 2.18	308.34 ± 6.27	4.90 ± 1.88
10	266.33 ± 3.07	7.60 ± 0.92	274.73 ± 4.83	8.61 ± 1.45
20	228.90 ± 10.57	11.23 ± 3.18	240.51 ± 5.25	9.14 ± 1.58
30	172.53 ± 2.32	21.71 ± 0.70	222.35 ± 2.24	13.01 ± 0.67
40	158.16 ± 7.45	19.37 ± 2.24	201.42 ± 0.13	15.41 ± 0.04
50	175.08 ± 21.98	22.20 ± 6.60	203.01 ± 0.26	15.81 ± 0.08

## Data Availability

All data and materials are available on request from the corresponding author. The data are not publicly available due to ongoing research using a part of the data.

## Supplementary Materials

The following supporting information can be downloaded at https://www.mdpi.com/article/10.3390/polym16192765/s1, Table S1: Dynamic viscosities of microemulsions with different Ozoile concentrations (0–50 wt%) prepared by high-intensity ultrasound (HIU) method. Average data obtained from measurements by rotating cylinder viscometer in triplicates; Table S2: VPTT, T_onset_, and ∆H of DRAP microspheres containing Ozoile (0–50 wt%). Data obtained by differential scanning calorimetry (DSC) analysis; Table S3: VPTT, T_onset_, and ∆H of DRAP microspheres containing Ozoile (0–50 wt%) with chitosan coating. Data obtained by differential scanning calorimetry (DSC) analysis.
