# Supplementary material for: Dual-Responsive Alginate/PNIPAM Microspheres Fabricated by Microemulsion-Based Electrospray
Source: Polymers (Basel). 2024 Sep 30;16(19):2765. doi: 10.3390/polym16192765 (PMC11478996; doi:10.3390/polym16192765)
Supplement: Supplementary file 1 [file polymers-16-02765-s001.zip › polymers-3161428-supplementary.pdf]

Supplementary Materials

Dual-Responsive Alginate/PNIPAM Microspheres Fabricated by Microemulsion-Based Electrospray

Gianluca Ciarleglio[1,2], Monica Placido[1], Elisa Toto[1], Maria Gabriella Santonicola[1,*]

[1] Department of Chemical Engineering Materials Environment, Sapienza University of Rome, Via del Castro Laurenziano 7, 00161 Rome, Italy;

[2] Erbagil s.r.l., Via Luigi Settembrini 13, 82037 Telese Terme, Italy

*Correspondence: mariagabriella.santonicola@uniroma1.it

Table S1. Dynamic viscosities of microemulsions with different Ozoile concentrations (0 – 50 wt%) prepared by high-intensity ultrasound (HIU) method. Average data obtained from measurements by rotating cylinder viscometer in triplicates.

Ozoile (wt%)	Dynamic viscosity (mPa·s)
0	54.8 ± 0.3
10	130.0 ± 0.5
20	152.9 ± 0.1
30	188.2 ± 1.7
40	219.0 ± 0.2
50	3117.0 ± 0.2

Table S2. VPTT, T_{onset}, and ΔH of DRAP microspheres containing Ozoile (0 – 50 wt%). Data obtained by differential scanning calorimetry (DSC) analysis.

Ozoile (wt%)	T_{onset} heating (°C)	VPTT heating (°C)	ΔH heating (J/g)	T_{onset} cooling (°C)	VPTT cooling (°C)	ΔH cooling (J/g)
0	36.92 ± 0.22	37.55 ± 0.22	0.46 ± 0.03	34.40 ± 0.18	33.61 ± 0.17	-0.50 ± 0.04
10	39.20 ± 0.42	39.54 ± 0.44	0.12 ± 0.03	36.99 ± 0.09	36.38 ± 0.08	-0.09 ± 0.00
20	39.59 ± 0.53	39.98 ± 0.47	0.10 ± 0.01	37.06 ± 0.18	36.44 ± 0.21	-0.10 ± 0.02
30	39.23 ± 0.15	39.94 ± 0.16	0.25 ± 0.02	37.29 ± 0.19	36.51 ± 0.19	-0.24 ± 0.02
40	38.87 ± 0.08	39.54 ± 0.14	0.06 ± 0.02	36.64 ± 0.41	35.79 ± 0.77	-0.06 ± 0.00
50	32.77 ± 0.40	33.46 ± 0.50	0.06 ± 0.05	-	-	-

Table S3. VPTT, T_{onset}, and ΔH of DRAP microspheres containing Ozoile (0 – 50 wt%) with chitosan coating. Data obtained by differential scanning calorimetry (DSC) analysis.

Ozoile (wt%)	T_{onset} heating (°C)	VPTT heating (°C)	ΔH heating (J/g)	T_{onset} cooling (°C)	VPTT cooling (°C)	ΔH cooling (J/g)
0	39.51 ± 0.18	39.87 ± 0.18	0.48 ± 0.24	39.51 ± 0.18	36.30 ± 0.20	-0.31 ± 0.07
10	39.45 ± 0.20	40.09 ± 0.26	0.05 ± 0.00	36.96 ± 0.03	36.35 ± 0.05	-0.05 ± 0.01
20	39.76 ± 0.73	40.49 ± 0.81	0.11 ± 0.03	37.07 ± 0.09	36.44 ± 0.07	-0.08 ± 0.02
30	39.10 ± 0.20	39.72 ± 0.19	0.14 ± 0.01	36.83 ± 0.20	36.17 ± 0.18	-0.12 ± 0.02
40	39.36 ± 0.20	40.01 ± 0.20	0.02 ± 0.00	37.68 ± 1.49	36.21 ± 0.06	-0.02 ± 0.01
50	-	-	-	-	-	-
